# Combined influence of media use on subjective health in elementary school children in Japan: a population-based study

**DOI:** 10.1186/1471-2458-12-432

**Published:** 2012-06-13

**Authors:** Harunobu Nakamura, Kumiko Ohara, Katsuyasu Kouda, Yuki Fujita, Tomoki Mase, Chiemi Miyawaki, Yoshimitsu Okita, Tetsuya Ishikawa

**Affiliations:** 1 Graduate School of Human Development and Environment, Kobe University, 3-11 Tsurukabuto, Nada, Kobe, Japan; 2 Department of Public Health, Kinki University Faculty of Medicine, 377-2 Ohno-higashi, Osaka-sayama, Japan; 3 Department of Childhood Education, Nagoya Women's University, 1302 Takamiya, Tenpaku, Nagoya, Japan; 4 Graduate School of Science and Technology, Shizuoka University, 3-5-1 Johoku, Naka, Hamamatsu, Japan

**Keywords:** Children, Media, Lifestyle, Subjective health

## Abstract

****Background**:**

In recent years in Japan, electronic games, home computers, and the internet have assumed an important place in people’s lives, even for elementary school children. Subjective health complaints have also become a problem among children. In the present study, we investigated the relationship between media use and health status in elementary school children in Japan.

****Methods**:**

A cross-sectional school-based population survey was conducted in 2009 with a sample of fourth-, fifth-, and sixth-grade children (age range: 10–12 years old) in elementary schools in Japan (n = 3,464). Self-reported health, lifestyle habits, and time spent using media were assessed.

****Results**:**

The use of games, television, and personal computers was significantly associated with lifestyle (p < 0.05) and subjective health (p < 0.05). In addition, the use of games, the use of television, and the use of personal computers were mutually associated. The greater the number of media used for more than 1 hour was, the higher the odds ratio of the association of media use with unhealthy lifestyle and subjective health complaints was. The plural use of these media had stronger associations with unhealthy lifestyle and subjective health complaints.

****Conclusions**:**

Game, television, and personal-computer use were mutually associated, and the plural use of these media had stronger associations with unhealthy lifestyle and subjective health complaints. Excessive use of media might be a risk for unhealthy lifestyle and subjective health complaints.

## **Background**

In recent years, innovation in electronic media technologies has progressed dramatically, and electronic games, home computers, and the internet have assumed an important place in the lives of both adults and children, even elementary school children. Rideout et al. reported that the time spent using television (TV), personal computers (PC), and video games has increased from 1999 through 2009 in the United States of America [1]. Olds et al. reported that Australian children aged 10–13 years engage in nearly 4 hours of media use per day [2]. In Japan, the use of electronic media is similar to that in other industrial countries. For example, the mean prevalence rates of televisions and PCs per household were 99.6 % and 76.0 %, respectively, in Japan in 2011 [3]. These media provide us with useful information and fulfilment, but the excessive use of them has been identified as a health issue, even for elementary school children. Extensive television viewing tends to be associated with obesity [4-6], sleep problems [7,8], and attention disorders [9]. Prolonged video viewing is related with poor attention span, poor creative imagination, and poor visual memory [10].

Subjective health encompasses the physical, emotional, mental, social and behavioral components of well-being and functioning as perceived by the individual [11-13], and subjective health complaints have become a problem among children. For example, Gobina et al. reported that health complaints, such as headache, stomach-ache, difficulties in getting to sleep, and nervousness were common among adolescents (11-, 13-, and 15-year-old boys and girls) in 19 countries in Europe and in the USA [14]. Ravens-Sieberer et al. reported that 44 % of school children have multiple recurrent health complaints, such as headache, stomach-ache, nervousness, sleeping difficulties, and dizziness, in 41 European and North-American countries. They also reported that older adolescents and girls had more health problems, and the gender difference increased with age [15].

The relation between media use and some aspects of subjective health, such as sleep problems, has been reported [7,8]. However, the relation has not been fully elucidated. Since both subjective health complaints and media use are common in adolescents, it is important to determine the relation between media use and the subjective health from a public health perspective. In addition, questions also arise regarding how much media use affects subjective health and what is the effect of the combination of media. However, little evidence has been collected on the influence of media use on children’s subjective health.

Therefore, we conducted a population-based cross-sectional study to investigate the current status of media use and the association of media use with health status and related factors in elementary school children in Japan.

## **Methods**

### **Participants**

We conducted a population-based cross-sectional study in three neighbouring cities (Awaji, Sumoto, and Minami-awaji) located in the urban area of Hyogo prefecture in the central part of Japan. A self-administered questionnaire was conducted in May 2009. The subjects were fourth-, fifth-, and sixth-graders (age range: 10–12 years old; mean ± standard deviation of age: 10.05 ± 0.81 years old) in public elementary schools in these three cities (n = 3,690). The responders included 3,464 children, and the response rate was 93.9 %. The participants included 1,056 fourth-graders (509 boys and 547 girls), 1,195 fifth-graders (597 boys and 598 girls), and 1,213 sixth-graders (615 boys and 598 girls). The study was approved by Human Ethics Committee of Graduate School of Human Development and Environment, Kobe University.

### **Questionnaire**

The questionnaire was anonymous and had three parts and a total of 21 fixed-choice questions. The first part included questions pertaining to the time spent using media (game, TV, and PC). Response options included:" none", " up to 1 hr", "up to 2 hrs", "2 hrs or more". The second part included 9 items pertaining to lifestyle, including the following: "bedtime", "waking time", "good feeling on waking", "breakfast eating", "eating breakfast alone", "dinner time", "eating dinner alone", "learning time at home", and "reading at home". The responses for "bedtime" included "-21:00", "21:00–22:00", "22:00–23:00", "23:00–24:00", "24:00-", and "irregular". The response for "waking time" included "-6:00", "6:00–7:00", "7:00–8:00", "8:00-", and "irregular". The responses for "good feeling on walking", "eating breakfast alone", "eating dinner alone", or "reading at home" included "usually", "often", "occasionally", and "seldom". The responses for "breakfast eating" included "everyday", "often", "occasionally", and "seldom". The responses for "dinner time" included "-18:00", "18:00–19:00", "19:00–20:00", "20:00–21:00", and "21:00-". The responses for "learning time at home" included "none", "up to 30 min", "30-60 min", "1-2 hrs", and "2 hrs-". The third part included 9 items pertaining to subjective health complaints, as follows: "depression", "sleeplessness", "ill at ease", "dizziness", "poor appetite", "headache", "abdominal pain", "short-tempered", and "negative thinking". Each item had 4 responses: "usually", "often", "occasionally", and "seldom". The questionnaire was distributed during class by a teacher at each elementary school, and then collected after it was completed.

### **Statistical analysis**

To assess the differences among factors, Student’s t test and a chi-square test (or Fisher’s exact test when appropriate) were performed. Logistic regression analysis was used to evaluate the association between the combined influence of media use and lifestyle habits or subjective health complaints, adjusting for gender and grade. Odds ratios (ORs) and 95 % confidence intervals (95 % CIs) were calculated. Differences with p-values <0.05 were considered significant. Statistical analysis was performed by SPSS® 18.0 J for Windows (SPSS Inc., Chicago, IL).

## **Results**

The amounts of time spent using games, TV, and PCs are shown in Table 1. The time spent using games was significantly less in girls than boys (p < 0.001). In addition, children in older grades spent more time using TV and PCs than children in younger grades (TV, p < 0.001; PCs, p < 0.001).

**Table 1:** Descriptive status of media use in elementary school children

		**Boys**			**Girls**			**p value gender / grades**
		**4th grade**	**5th grade**	**6th grade**	**4th grade**	**5th grade**	**6th grade**	
Game	None	86	91	98	167	195	186	* / ns
	Up to 1 hr	289	311	315	328	346	337	
	Up to 2 hrs	85	136	134	42	38	53	
	2 hrs or more	44	51	65	8	14	18	
	Total	504	589	612	545	593	594	
TV	None	33	33	25	36	26	20	ns / *
	Up to 1 hr	156	170	164	201	178	148	
	Up to 2 hrs	147	178	187	151	178	146	
	2 hrs or more	160	200	226	140	198	275	
	Total	496	581	602	528	580	589	
PC	None	324	331	338	368	332	301	ns / *
	Up to 1 hr	150	208	216	150	212	228	
	Up to 2 hrs	14	26	35	15	26	43	
	2 hrs or more	12	14	15	2	9	14	
	Total	500	579	604	535	579	586	

Values are head-counts. TV: television, PC: personal computer.

*p < 0.05 for chi-square analysis.

Lifestyle according to time spent using games, TV, or PCs is shown in Table 2. Children who spent more than 1 hr using games had significantly later bedtimes than those who spent less than 1 hr using games (p < 0.001) and had later waking times (p < 0.001), a lower rate of "good feeling on waking" (p < 0.001), a lower frequency of eating breakfast (p < 0.001), a higher frequency of eating breakfast alone (p < 0.001), had less learning time at home (p < 0.001), and spent less time reading at home (p < 0.001). Those who spent more than 1 hr using TV had significantly later bedtimes than those who spent less than 1 hr using TV (p < 0.001) and had later waking times (p = 0.002), a lower rate of "good feeling on waking" (p < 0.001), a lower frequency of eating breakfast (p = 0.002), a higher frequency of eating breakfast alone (p = 0.001), later dinner times (p = 0.005), and spent less time reading at home (p < 0.001). Those who spent more than 1 hr using PCs had significantly later bedtimes than those who spent less than 1 hr using PCs (p < 0.001) and had later waking times (p = 0.025), a lower rate of "good feeling on waking" (p = 0.005), a lower frequency of eating breakfast (p < 0.001), a higher frequency of "eating breakfast alone" (p < 0.001), a higher frequency of "eating dinner alone" (p = 0.005), and had more "learning time at home" (p = 0.001).

**Table 2:** Comparative analysis of media use and lifestyle

	**Game**		**TV**		**PC**	
	**less than 1 hr**	**more than 1 hr**	**less than 1 hr**	**more than 1 hr**	**less than 1 hr**	**more than 1 hr**
Bedtime						
−21:00	91	13*	57	43*	99	4*
21:00–22:00	817	118	409	511	892	30
22:00–23:00	964	227	366	808	1090	84
23:00–24:00	210	80	66	217	250	37
24:00-	22	24	10	35	33	11
Irregular	633	226	278	564	782	58
Total	2737	688	1186	2178	3146	224
Waking time						
−6:00	191	30*	97	119*	202	15*
6:00–7:00	1327	287	580	1003	1490	98
7:00–8:00	944	265	379	814	1116	74
8:00-	33	15	15	32	38	8
Irregular	234	90	114	202	295	27
Total	2729	687	1185	2170	3141	222
Good feeling on waking						
Usually	727	115*	356	468*	784	41*
Often	1450	316	568	1170	1637	108
Occasionally	432	183	207	400	551	52
Seldom	89	56	38	102	127	16
Total	2698	670	1169	2140	3099	217
Breakfast eating						
Everyday	2455	554*	1069	1886*	2783	180*
Often	194	64	74	179	229	21
Occasionally	58	42	24	74	87	13
Seldom	21	23	8	34	33	10
Total	2728	683	1175	2173	3132	224
Eating breakfast alone						
Usually	353	107*	130	321*	408	41*
Often	440	107	175	361	494	41
Occasionally	471	154	212	408	570	54
Seldom	1457	310	658	1075	1655	85
Total	2721	678	1175	2165	3127	221
Dinner time						
−18:00	101	24	60	63*	114	10
18:00–19:00	730	188	342	562	855	46
19:00–20:00	1383	332	564	1119	1577	112
20:00–21:00	440	114	185	358	500	44
21:00-	54	21	26	50	69	7
Total	2708	679	1177	2152	3115	219
Eating dinner alone						
Usually	68	23	31	59	78	12*
Often	235	65	93	202	278	21
Occasionally	425	127	180	364	494	49
Seldom	1980	460	867	1528	2261	138
Total	2708	675	1171	2153	3111	220
Learning time at home						
none	78	42*	44	72	103	15*
up to 30 min	776	211	358	599	901	63
30–60 min	1264	282	528	1000	1444	83
1–2 hrs	491	120	198	406	554	50
2 hrs-	110	29	50	88	124	11
Total	2719	684	1178	2165	3126	222
Reading at home						
Usually	341	55*	172	218*	364	30
Often	1140	212	494	831	1245	80
Occasionally	755	221	328	634	907	55
Seldom	492	195	186	487	618	58
Total	2728	683	1180	2170	3134	223

Values are head-counts. TV: television, PC: personal computer.

*p<0.05for chi-square test.

Subjective health complaints according to time spent using games, TV, and PCs are shown in Table 3. Children who spent more than 1 hr using games had a significantly higher frequency of depression than those who spent less than 1 hr using games (p < 0.001) and had a higher frequency of sleeplessness (p < 0.001), feeling ill at ease (p < 0.001), dizziness (p < 0.001), poor appetite (p < 0.001), headache (p = 0.002), abdominal pain (p = 0.003), being short-tempered (p < 0.001), and negative thinking (p < 0.001). Those who spent more than 1 hr using TV had a significantly higher frequency of sleeplessness than those who spent less than 1 hr using TV (p = 0.032) and had a higher frequency of feeling ill at ease (p = 0.001), poor appetite (p = 0.037), and being short-tempered (p < 0.001). Those who spent more than 1 hr using PCs had a significantly higher frequency of depression than those who spent less than 1 hr using PCs (p = 0.001) and had a higher frequency of sleeplessness (p = 0.022), feeling ill at ease (p = 0.008), dizziness (p < 0.001), poor appetite (p = 0.003), headache (p = 0.001), abdominal pain (p = 0.010), being short-tempered (p < 0.001), and negative thinking (p < 0.001).

**Table 3:** Comparative analysis of media use and subjective health complaints

	**Game playing**		**TV viewing**		**PC operating**	
	**less than 1 hr**	**more than 1 hr**	**less than 1 hr**	**more than 1 hr**	**less than 1 hr**	**more than 1 hr**
Depression						
Usually	173	82*	77	173	217	33*
Often	617	189	293	496	739	55
Occasionally	898	195	361	712	1006	61
Seldom	1052	220	458	798	1186	76
Total	2740	686	1189	2179	3148	225
Sleeplessness						
Usually	282	118*	131	263*	360	37*
Often	710	209	320	585	847	57
Occasionally	745	181	291	615	835	68
Seldom	986	173	435	705	1087	60
Total	2723	681	1177	2168	3129	222
Ill at ease						
Usually	371	153*	173	343*	469	48*
Often	593	179	238	514	702	53
Occasionally	788	187	327	631	900	61
Seldom	965	156	439	664	1051	54
Total	2717	675	1177	2152	3122	216
Dizziness						
Usually	125	58*	67	109	160	20*
Often	385	117	161	333	441	51
Occasionally	543	130	211	454	625	43
Seldom	1649	372	728	1259	1880	108
Total	2702	677	1167	2155	3106	222
Poor appetite						
Usually	141	50*	59	127*	173	l16*
Often	561	168	231	490	657	62
Occasionally	840	244	364	704	990	78
Seldom	1153	216	506	835	1281	64
Total	2695	678	1160	2156	3101	220
Headache						
Usually	253	87*	129	204	297	36*
Often	738	206	315	608	856	71
Occasionally	833	206	342	688	965	61
Seldom	884	179	388	651	993	53
Total	2708	678	1174	2151	3111	221
Abdominal pain						
Usually	162	58*	73	145	196	24*
Often	449	139	201	376	532	45
Occasionally	721	176	302	577	823	60
Seldom	1354	298	582	1041	1538	89
Total	2686	671	1158	2139	3089	218
Short-tempered						
Usually	406	151*	172	378*	493	61*
Often	641	192	246	569	764	53
Occasionally	848	199	360	670	967	65
Seldom	795	132	377	535	867	42
Total	2690	674	1155	2152	3091	221
Negative thinking						
Usually	257	87*	118	222	297	42*
Often	712	208	304	592	840	60
Occasionally	1005	243	418	806	1149	76
Seldom	748	145	339	544	842	46
Total	2722	683	1179	2164	3128	224

Values are head-counts. TV: television, PC: personal computer.

*p<0.05for chi-square test.

The relationships among games, TV, and PCs are shown in Table 4. Games were positively related with TV (Spearman’s ρ = 0.191, p < 0.001) or PCs (Spearman’s ρ = 0.242, p < 0.001). TV was positively related with PCs (Spearman’s ρ = 0.110, p < 0.001).

**Table 4:** Correlation coefficients among game, TV, and PC use

	**Game**	**TV**	**PC**
Game	-	0.191*	0.242*
TV		-	0.110*
PC			-

The number is Spearman’s ρ. TV: television, PC: personal computer.

*p < 0.05 for Spearman’s correlation coefficients.

The results of the logistic regression model exploring the association between the plural use of media and lifestyle are shown in Table 5. Children who spent more than 1 hr on each of one, two, or all media devices were almost twice as likely (odds of one device: 1.224, p = 0.034; odds of two devices: 1.908, p < 0.001; odds of all devices: 2.258, p < 0.001) to go to bed at later hours, after the model was adjusted for sex and grade. Those who spent more than 1 hr on each of all media devices were almost two and one-half times more likely (odds: 2.502, p = 0.001) to wake at later hours, after the model was adjusted for sex and grade. Those who spent more than 1 hr on each of two or all media devices were twice as likely (odds of two devices: 2.071, p < 0.001) or two and one-half times as likely (odds of all devices: 2.621, p < 0.001) to have a bad feeling on waking, after the model was adjusted for sex and grade. Those who spent more than 1 hr on each of two or all media devices were about 4 times (odds of two devices: 3.843, p < 0.001) or 8 times (odds of all devices: 7.816, p < 0.001) more likely to eat breakfast with low frequency, after the model was adjusted for sex and grade. Those who spent more than 1 hr on each of one, two, or all media devices were about one and one-half times more likely (odds of one device: 1.301, p = 0.004; odds of two devices: 1.436, p = 0.003; odds of all devices: 1.572, p = 0.046) to eat breakfast alone, after the model was adjusted for sex and grade. Those who spent more than 1 hr on each of two or all media devices were about one and one-half times more likely (odds of two devices: 1.436, p = 0.003; odds of all devices: 1.572, p = 0.046) to eat breakfast alone, after the model was adjusted for sex and grade. Those who spent more than 1 hr on each of two media devices were almost one and one-half times more likely (odds: 1.306, p = 0.049) to eat dinner at later hours, after the model was adjusted for sex and grade. Those who spent more than 1 hr on each of one, two, or all media devices were about one and one-half times (odds of one device: 1.360, p < 0.001; odds of all devices: 1.633, p = 0.025) or two times (odds of two devices: 1.835, p < 0.001) more likely to read books with low frequency, after the model was adjusted for sex and grade.

**Table 5:** Associations between plural use of media and lifestyles

	**not adjusted**			adjusted*		
	**Odds**	**95 % CI**	**p value**	**Odds**	**95 % CI**	**p value**
Bedtime						
all less than 1 hr (ref)	1			1		
one - more than 1 hr	1.158	(0.963-1.393)	0.119	1.224	(1.015-1.476)	0.034
two- more than 1 hr	1.871	(1.484-2.360)	<0.001	1.908	(1.502-2.424)	<0.001
all more than 1 hr	2.014	(1.302-3.117)	0.002	2.258	(1.446-3.525)	<0.001
Waking time						
all less than 1 hr (ref)	1			1		
one - more than 1 hr	1.038	(0.800-1.346)	0.779	1.088	(0.837-1.415)	0.527
two– more than 1 hr	1.325	(0.954-1.842)	0.093	1.286	(0.918-1.801)	0.144
all more than 1 hr	2.328	(1.366-3.967)	0.002	2.502	(1.452-4.312)	0.001
Good awakened feeling						
all less than 1 hr (ref)	1			1		
one - more than 1 hr	1.123	(0.920-1.370)	0.256	1.107	(0.906-1.352)	0.319
two– more than 1 hr	2.155	(1.690-2.747)	<0.001	2.071	(1.617-2.652)	<0.000
all more than 1 hr	2.754	(1.762-4.304)	<0.001	2.621	(1.671-4.111)	<0.000
Breakfast eating						
all less than 1 hr (ref)	1			1		
one - more than 1 hr	1.401	(0.853-2.301)	0.182	1.406	(0.855-2.311)	0.179
two– more than 1 hr	4.168	(2.501-6.945)	<0.001	3.843	(2.286-6.462)	<0.001
all more than 1 hr	8.305	(4.222-16.339)	<0.001	7.816	(3.935-15.525)	<0.000
Eating breakfast alone						
all less than 1 hr (ref)	1			1		
one - more than 1 hr	1.315	(1.101-1.570)	0.002	1.301	(1.089-1.555)	0.004
two– more than 1 hr	1.489	(1.181-1.877)	0.001	1.436	(1.135-1.818)	0.003
all more than 1 hr	1.647	(1.059-2.563)	0.027	1.572	(1.007-2.452)	0.046
Dinner eating						
all less than 1 hr (ref)	1			1		
one - more than 1 hr	0.982	(0.800-1.206)	0.865	0.979	(0.797-1.202)	0.839
two– more than 1 hr	1.329	(1.024-1.725)	0.033	1.306	(1.001-1.703)	0.049
all more than 1 hr	1.069	(0.624-1.830)	0.808	1.048	(0.610-1.801)	0.865
Eating dinner alone						
all less than 1 hr (ref)	1			1		
one - more than 1 hr	1.227	(0.952-1.582)	0.115	1.220	(0.946-1.575)	0.126
two– more than 1 hr	1.222	(0.873-1.711)	0.242	1.154	(0.820-1.624)	0.412
all more than 1 hr	1.657	(0.920-2.985)	0.092	1.563	(0.864-2.828)	0.140
Learning time at home						
all less than 1 hr (ref)	1			1		
one - more than 1 hr	0.923	(0.763-1.116)	0.408	0.949	(0.784-1.149)	0.591
two– more than 1 hr	0.957	(0.740-1.236)	0.735	0.908	(0.755-1.273)	0.883
all more than 1 hr	0.629	(0.397-0.997)	0.049	0.672	(0.423-1.069)	0.094
Reading at home						
all less than 1 hr (ref)	1			1		
one - more than 1 hr	1.356	(1.157-1.588)	<0.001	1.360	(1.159-1.596)	<0.001
two– more than 1 hr	2.081	(1.679-2.580)	<0.001	1.835	(1.474-2.285)	<0.001
all more than 1 hr	1.807	(1.184-2.756)	0.006	1.633	(1.065-2.504)	0.025

ref: reference.

*adjusted by gender and grade.

The results of the logistic regression model exploring the association between the plural use of media and subjective complaints are shown in Table 6. Children who spent more than 1 hr on each of two or all media devices were almost one and one-half times more likely (odds of two devices: 1.456, p = 0.001; odds of all devices: 1.644, p = 0.024) to feel "depression", after the model was adjusted for sex and grade. Those who spent more than 1 hr on each of two or all media devices were almost one and one-half times (odds of two devices: 1.477, p = 0.001) or two times (odds of all devices: 1.783, p = 0.007) more likely to feel "sleeplessness", after the model was adjusted for sex and grade. Those who spent more than 1 hr on each of one, two, or all media devices were between 1.2 and 2.5 times more likely (odds of one device: 1.286, p = 0.003; odds of two devices: 1.704, p < 0.001; odds of all devices: 2.476, p < 0.001) to feel "ill at ease", after the model was adjusted for sex and grade. Those who spent more than 1 hr on each of two or all media devices were almost two times more likely (odds of two devices: 1.728, p < 0.001; odds of all devices: 1.791, p = 0.016) to feel "dizziness", after the model was adjusted for sex and grade. Those who spent more than 1 hr on each of one or two media devices were almost one and one-half times more likely (odds of one device:1.248, p = 0.018; odds of two devices: 1.555, p < 0.001) to have a "poor appetite", after the model was adjusted for sex and grade. Those who spent more than 1 hr on each of two or all media devices were almost one and one-half times (odds of two devices: 1.327, p = 0.012) or two times (odds of all devices: 1.854, p = 0.004) more likely to suffer from "headache", after the model was adjusted for sex and grade. Those who spent more than 1 hr on each of two media devices were almost one and one-half times more likely (odds: 1.420, p = 0.005) to feel "abdominal pain", after the model was adjusted for sex and grade. Those who spent more than 1 hr on each of one, two, or all media devices were almost one and one-half times (odds of one device:1.446, p < 0.001) or two times (odds of two devices: 2.271, p < 0.001; odds of all devices: 1.864, p = 0.004) more likely to feel "short-tempered", after the model was adjusted for sex and grade. Those who spent more than 1 hr on each of two or all media devices were almost one and one-half times (odds of two devices: 1.463, p = 0.001) or two times (odds of all devices: 2.128, p < 0.001) more likely to experience "negative thinking", after the model was adjusted for sex and grade.

**Table 6:** Associations between plural use of media and subjective health complaints

	**not adjusted**			adjusted*		
	**Odds**	**95 % CI**	**p value**	**Odds**	**95 % CI**	**p value**
Depression						
all less than 1 hr (ref)	1			1		
one - more than 1 hr	1.132	(0.952-1.345)	0.160	1.139	(0.958-1.355)	0.139
two- more than 1 hr	1.472	(1.176-1.842)	0.001	1.456	(1.158-1.830)	0.001
all more than 1 hr	1.639	(1.068-2.518)	0.024	1.644	(1.067-2.532)	0.024
Sleeplessness						
all less than 1 hr (ref)	1			1		
one - more than 1 hr	1.037	(0.881-1.220)	0.664	1.074	(0.911-1.265)	0.395
two - more than 1 hr	1.422	(1.147-1.762)	0.001	1.477	(1.185-1.840)	0.001
all more than 1 hr	1.631	(1.073-2.478)	0.022	1.783	(1.168-2.721)	0.007
Ill at ease						
all less than 1 hr (ref)	1			1		
one - more than 1 hr	1.233	(1.045-1.454)	0.013	1.286	(1.087-1.521)	0.003
two - more than 1 hr	1.848	(1.488-2.297)	<0.001	1.704	(1.362-2.131)	<0.001
all more than 1 hr	2.452	(1.600-3.757)	<0.001	2.476	(1.601-3.829)	<0.001
Dizziness						
all less than 1 hr (ref)	1			1		
one - more than 1 hr	0.929	(0.758-1.138)	0.475	0.912	(0.744-1.119)	0.378
two - more than 1 hr	1.622	(1.264-2.081)	<0.001	1.728	(1.338-2.232)	<0.001
all more than 1 hr	1.740	(1.091-2.775)	0.020	1.791	(1.117-2.872)	0.016
Poor appetite						
all less than 1 hr (ref)	1			1		
one - more than 1 hr	1.235	(1.030-1.482)	0.023	1.248	(1.040-1.498)	0.018
two - more than 1 hr	1.576	(1.246-1.994)	<0.001	1.555	(1.224-1.975)	<0.001
all more than 1 hr	1.497	(0.954-2.349)	0.079	1.505	(0.956-2.369)	0.077
Headache						
all less than 1 hr (ref)	1			1		
one - more than 1 hr	0.950	(0.807-1.119)	0.539	0.973	(0.826-1.146)	0.742
two - more than 1 hr	1.258	(1.013-1.561)	0.038	1.327	(1.064-1.654)	0.012
all more than 1 hr	1.713	(1.125-2.607)	0.012	1.854	(1.214-2.833)	0.004
Abdominal pain						
all less than 1 hr (ref)	1			1		
one - more than 1 hr	1.016	(0.842-1.226)	0.871	1.033	(0.855-1.248)	0.735
two - more than 1 hr	1.394	(1.095-1.775)	0.007	1.420	(1.110-1.817)	0.005
all more than 1 hr	1.443	(0.905-2.301)	0.123	1.506	(0.941-2.411)	0.088
Short-tempered						
all less than 1 hr (ref)	1			1		
one - more than 1 hr	1.409	(1.195-1.661)	<0.001	1.446	(1.225-1.706)	<0.001
two - more than 1 hr	2.186	(1.759-2.716)	<0.001	2.271	(1.819-2.836)	<0.001
all more than 1 hr	1.738	(1.140-2.651)	0.010	1.864	(1.218-2.853)	0.004
Negative thinking						
all less than 1 hr (ref)	1			1		
one - more than 1 hr	1.049	(0.890-1.236)	0.566	1.069	(0.906-1.261)	0.432
two - more than 1 hr	1.339	(1.078-1.662)	0.008	1.463	(1.172-1.826)	0.001
all more than 1 hr	1.922	(1.264-2.921)	0.002	2.128	(1.394-3.250)	<0.001

ref: reference.

*adjusted by gender and grade.

## **Discussion**

We investigated the relationship between media use and lifestyle or subjective health complaints in elementary school children in Japan. The main finding of the present study was that those who spent more time using media had less healthy lifestyles and more subjective health complaints. In addition, game use, TV use, and PC use were mutually associated, and the plural use of these media had a stronger association with unhealthy lifestyles and subjective health complaints.

In the present study, those who spent more than 1 hr using a media device went to bed at a later time, woke at a later time, had a low frequency of feeling good on waking, ate breakfast with low frequency, and ate breakfast alone with high frequency. These indicators related to sleep or breakfast are associated with lifestyle regularity. According to the previous study, there was some evidence indicating that media use consumed time or displaced other activities after school. Van den Bulck reported that secondary school students who watched more television, played computer games, or used the internet went to bed later [16], and skipped meals [17,18]. These results suggest that media use may be responsible for lifestyle irregularity through the displacement of daily activities or through surplus time spent.

Regarding subjective health complaints, those who spent more than 1 hr using a media device were more likely to have subjective health complaints, regardless of the kind of media. Bener et al. and Mathers et al. showed similar results in 6- to 18-year-old children and adolescents (mean age, 16 years old) [19,20]. Namely, they found that spending prolonged hours using a computer or TV was associated with poor life habits, such as sleeplessness [19,20]. Nagane et al. reported that lifestyle irregularity is associated with health complaints in university students [21]. In the present study, excessive media use was related with unhealthy lifestyles, which may be causally related to the frequency of subjective health complaints. In addition, the present results showed that game and PC use had stronger associations with multiple subjective health complaints than TV viewing, although the prevalence of the excessive use of TV was greater than that for games or PCs. The reason for this discrepancy among these media is not clear. There have been few previous reports in which differences among media are discussed. The present study also cannot address the reason for this discrepancy. It has been reported in children and adolescents that TV viewing is related with sedentary behavior and obesity because it involves long periods of sitting [19,22,23]. On the other hand, most of the studies regarding addiction referred to the influence of game or computer use [24-27]. It is not clear why specific health issues are connected to specific media. The results of the present and previous studies may reflect the mechanism underlying the related health issues. Indeed, games, TV, and PCs involve very different communication methods. For example, TV watching is a one-way communication experience, while game-playing and PC operation involve two-way communication. In future studies, we should focus on the differences among these media.

Game use, TV use, and PC use for more than 1 hr per day were positively mutually correlated. We then verified the cumulative effects when these media were used for more than 1 hr combined. It was found that the greater the number of media used for more than 1 hr was, the higher the odds ratio of the association between media use and subjective health complaints was. Up to now, there has been little evidence with which to assess the combined effect of media on subjective health complaints in elementary school children. In adolescents, Punamaki et al. found that intensive usage of information and communication technology was associated with poor subjective health in adolescents [8]. In the present study, the plural use of media was also strongly associated with unhealthy lifestyles. This unhealthy lifestyle that accompanies the plural use of media may have an influence on subjective health complaints. As a consequence, the present results suggest that the plural use of media has a cumulative influence on subjective health.

The limitations of this study should be noted. First, the present study has a cross-sectional design which cannot draw conclusion about any cause-effect relationship. Second, the samples were collected from a limited area in Japan. Future studies will need to collect samples from a wider area and to employ a longitudinal design for the estimation of any cause-effect relationship. Third, the questionnaire in the present study has not been sufficiently validated, and the link between subjective health and objectively assessed health indices has not been established. In addition, we did not separate sedentary gaming from active gaming. Active gaming could be health enhancing, such as through energy expenditure [28]. Therefore, interpretation of our results may be limited.

## **Conclusions**

We investigated the relationship between media use and lifestyle or subjective health complaints in elementary school children in Japan. Media use was positively associated with unhealthy lifestyles and subjective health complaints. In addition, game, TV, and PC use were mutually associated, and the plural use of these media had stronger associations with unhealthy lifestyles and subjective health complaints.

## **Competing interests**

The authors declare that they have no competing interests.

## **Authors' contributions**

All authors were involved with the study design. HN, KO, and TI collected the data. HN, KK, and YF analyzed the data. HN drafted the manuscript with contributions from YO, TM, and CM. All authors read and approved the manuscript.

## Pre-publication history

The pre-publication history for this paper can be accessed here:

http://www.biomedcentral.com/1471-2458/12/432/prepub
